# Immunoprotection against mixed *Eimeria* spp. infections in goat kids induced by X-irradiated oocysts

**DOI:** 10.1007/s00436-022-07465-z

**Published:** 2022-03-03

**Authors:** Emilio Barba, Aránzazu Carmen Guedes, José Manuel Molina, Sergio Martín, María Carmen Muñoz, Otilia Ferrer, Pedro Carlos Lara, Carlos Hermosilla, Anja Taubert, Antonio Ruiz

**Affiliations:** 1 Parasitology Unit, Department of Animal Pathology, Faculty of Veterinary Medicine, University of Las Palmas de Gran Canaria, 35413 Arucas, Las Palmas Spain; 2 University Fernando Pessoa and University Hospital San Roque, Las Palmas, Spain; 3 Institute of Parasitology, Justus Liebig University Giessen, Biomedical Research Center Seltersberg, Giessen, Germany

**Keywords:** *Eimeria* spp., X-Rad attenuation, Immunoprotection, Goats, Vaccine

## Abstract

Strategies to control goat coccidiosis traditionally rely on the use of management practices combined with anticoccidial treatments, and limited effort has been made, so far, to address immunological control of caprine *Eimeria* infections. Previously, we showed that monospecific immunization with X-Rad-attenuated *Eimeria ninakohlyakimovae* oocysts induced considerable immunoprotection upon challenge. In the present study, we conducted a similar vaccination trial but using a mixture of caprine *Eimeria* species typically present in natural infected goats. For immunization, sporulated oocysts were attenuated by X irradiation (20 kilorad). All infections were performed orally applying 10^5^ sporulated oocysts of mixed *Eimeria* spp. per animal. In total, 18 goat kids were grouped as follows: (G1) immunized + challenge infected; (G2) primary + challenge infected; (G3) challenge infection control; and (G4) non-immunized/non-infected control. Overall, goat kids infected with attenuated oocysts (= immunized) shed less oocysts in the faeces and showed a lower degree of clinical coccidiosis than animals infected with non-attenuated oocysts. Animals of both challenge groups (G1 and G2) showed partial immunoprotection upon reinfection when compared to challenge infection control (G3). However, the degree of immunoprotection was less pronounced than recently reported for monospecific vaccination against *Eimeria ninakohlyakimovae*, most probably due to the complexity of the pathogenesis and related immune responses against mixed *Eimeria* spp. infections. Nevertheless, the data of the present study demonstrate that immunization with attenuated *Eimeria* spp. oocysts may be worth pursuing as a strategy to control goat coccidiosis.

## Introduction

Coccidiosis is considered one of the most prevalent and economically important parasitic diseases in goat production systems worldwide (Cavalcante et al. 2012; Fthenakis and Papadopoulos 2018; Windsor et al. 2018). Economic losses are mainly attributed to delayed growth rates and deaths of goat kids, which can sometimes be as high as 30% (Koudela and Boková 1998). The effects of coccidiosis usually extend from 4 to 10 weeks of age, and are especially important around weaning time due to changes in feeding and stress caused during this period (Ruiz et al. 2006). As a general characteristic of Eimeriosis, goats and other ruminants commonly experience multispecies infections with up to 7–8 different *Eimeria* species in the field, while monospecific infections hardly occur (Ruiz et al. 2006; Balicka-Ramisz et al. 2012; Alcala-Canto et al. 2020). Among 11 species recognized as most frequent in goats, *E. ninakohlyakimovae* and *E. arloingi* are considered the most pathogenic ones (Ruiz et al. 2013a, b; Silva et al. 2014a). The development and multiplication of these *Eimeria* species in the small and large intestine result in dysentery associated with diarrhoea of different consistency and colour, weakness, anorexia and dehydration that can occasionally lead to death of affected animals (Ruiz et al. 2013a, b; Silva et al. 2014a). Other species, such as *E. christenseni* and *E. caprina*, have also been described as moderately pathogenic (Taylor and Catchpole 1994).

Since coccidiosis is a faecal-oral transmitted disease, hygiene and general management measures are considered key tools for disease control (Smith and Sherman 2009; Chartier and Paraud 2012). These measures are usually combined with prophylactic or metaphylactic administration of anticoccidials (Daugschies and Najdrowski 2005). Although there is a wide range of products that have been shown to be effective against coccidial infections in ruminants (Keeton and Navarre 2018), their use is increasingly restricted by both, the growing emergence of anticoccidial resistance and European Union–based limitations on the use of drugs and additives in animal husbandry. Currently, *Eimeria*-related resistance to toltrazuril has recently been demonstrated in Norwegian sheep flocks (Odden et al. 2018). It would be therefore of interest the development of alternative control measures to complement management practices and drug-based treatments, for instance by immunoprophylactic approaches.

*Eimeria* infections are well known to induce protective immunity in livestock under natural conditions. In this regard, it has been demonstrated that *Eimeria* species of cattle (Hermosilla et al. 1999; Taubert et al. 2008), sheep (Daugschies and Najdrowski 2005; Zanetti Lopes et al. 2013) and goats (Ruiz et al. 2013a, 2014; Matos et al. 2017a, b) induce effective immunoprotection mechanisms, which prevent the development of clinical disease after challenge infections. However, immune reactions against ruminant coccidiosis are complex and involve both innate and acquired mechanisms including cellular and humoral responses (Taubert et al. 2008; Matos et al. 2017b, 2018). Besides, immunity against one *Eimeria* species is mainly species specific, and does not protect animals infected with other species (Khodakaram-Tafti and Hashemnia 2017).

So far, commercial vaccines have only been developed for poultry, which highly suffers in terms of animal welfare and economics from coccidiosis. Commercial vaccines include both, live and attenuated vaccines, the latter of which mainly relying on the establishment of precocious lines (Williams 2002; Jenkins et al. 2012; Milbradt et al. 2014). Anticoccidial live and attenuated vaccines both usually contain a cocktail of the most pathogenic *Eimeria* species that affect poultry (Crouch et al. 2003; McDonald and Shirley 2009). An enormous effort was made on the development of recombinant vaccines against avian coccidiosis; however, no recombinant anticoccidial vaccine is currently available on the market (Soutter et al. 2020).

Despite the success of anticoccidial vaccines in poultry industry, attempts to develop vaccines against ruminant *Eimeria* infections are very limited. We have reported that immunization with live but attenuated *E. ninakohlyakimovae* oocysts protects goat kids from clinical coccidiosis at a comparable level as natural primary infections (Ruiz et al. 2014). However, given that monospecific infections hardly occur in the field and that immunity against *Eimeria* species predominantly seems to be species-specific, the development of a vaccine that includes several caprine *Eimeria* species, particularly the most pathogenic ones, would be more appropriate.

In the current work, we performed a vaccination trial in goats applying a mixture of attenuated oocysts of different caprine *Eimeria* species and assessed its immunoprotective capacity towards a homologous challenge infection.

## Material and methods

### Animals

In total, 18 goats (Majorera breed) were included in the vaccination trial. They were purchased at 1–5 days of life from a local farm in the south of Gran Canaria. All animals were housed in sterilized cages for the total duration of the experiments; these cages were located in the Experimental Animal House of the Scientific and Technological Park (Veterinary Faculty) of the University of Las Palmas de Gran Canaria (Spain). In order to prevent prior infections with coccidia, goat kids were treated with Vecoxan® (Jansen Laboratories) and Halocur® (Intervet) upon arrival. To control for unwanted *Eimeria* infections, coprological analyses were repeatedly performed on goat kid faeces by conventional parasitological methods: upon arrival at the facility, 2 weeks later and weekly until the beginning of the experiment (week 5 of life: day 1). Goat kids were only included in the vaccination trial when deemed free of *Eimeria* infections. Animals were fed milk-replacer (Bacilactol®, Capisa) and starter feed (Capisa), and were provided with sterile hay, mineral salts and water ad libitum. All animal procedures were conducted in strict accordance with national ethics, the current European Animal Welfare Legislation (ART13TFEU) and by institutional review board-approved protocols.

### Parasites

Viable oocysts were obtained from faeces of animals naturally infected with *Eimeria* spp. and stored in bags at 4 °C until processing. For oocyst purification, we followed the method described by Hermosilla et al. (2002). Briefly, faecal material was washed with tap water and filtered with decreasing pore sizes to reduce detritus. The resulting suspension was dispensed in uncovered plastic receptacles containing saturated sugar solution (3 kg sugar/2 l water) at a ratio of 1:1. The containers were completely filled to form a meniscus and covered with glass plates to collect oocysts by flotation. Flotated oocysts were collected by washing them off the glass plates. To increase the yield, the mixture was stirred every 2 h and the glasses were replaced, for a total of 3 consecutive days. All washes were kept at 4 °C and concentrated by centrifugation at 3000 × *g* for 10 min. Oocyst sporulation was achieved by incubation at room temperature (25 °C) for 1 week in 2% potassium dichromate solution under constant stirring and air infusion into the suspension.

To estimate *Eimeria* species present in the inoculum, four samples were analysed microscopically at different days and, at each occasion, a total of 250 sporulated oocysts per sample were analysed. Only oocysts that had fully completed the sporulation process were included in the counting. Species differentiation was based on morphological criteria following the keys previously described by other authors (Levine and Ivens 1986; Alyousif et al. 1992; Soe and Pomroy 1992).

For attenuation, sporulated oocysts were subjected to a total irradiation of 20 kilorads with an intensity of 6 mV x-rays and a speed of 50 cGy/min for 15 min. Irradiation was performed in 25 cm^2^ (Nunc) culture flasks in a total volume of 20 ml oocyst solution using the Mevatron linear accelerator (Siemens, Germany) as the x-ray source.

### Experimental design (clinical, productive and parasitological analysis)

The animals were divided into four experimental groups: (i) animals immunized with attenuated oocysts at 5 weeks of age and re-infected 3 weeks later with non-attenuated oocysts (*n* = 5) (G1: immunized + challenge infected, reflecting induced immunity); (ii) animals infected at 5 weeks of age with non-attenuated oocysts and re-infected 3 weeks later with the same type of oocysts (*n* = 5) (G2: primary + challenge infected, reflecting natural immunity); (iii) primary infected animals with non-attenuated oocysts at 8 weeks of age (*n* = 4) (G3: challenge infection control); (iv) non-immunized, non-infected animals (*n*=4) (G4: non-immunized/non-infected control).

All infections were performed orally by a gastro-ruminal tube using an infective dose of 1×10^5^ oocysts of the *Eimeria* spp. mixture as described above, attenuated or non-attenuated. Immunoprotection levels were evaluated by the following measures: productive parameters (body weight), clinical parameters (presence of clinical signs, such as diarrhoea), parasitological parameters (faecal oocyst counts, OPG; *Eimeria* speciation). For the productive follow-up, the weight of the animals was controlled weekly, while for the evaluation of the clinical course of the disease, a daily examination of all animals was carried out paying special attention on faecal consistency using the following score: (1) normally formed faeces; (2) unformed faeces of light reduced consistency; (3) faeces of moderate reduced consistency; (4) yellowish, greenish or brownish liquid faeces; (5) liquid faeces of reddish colour and/or presence of mucosal pieces.

Faecal samples were taken rectally on a daily basis for parasitological determinations, starting 14 days after infection (d. p. i.). All samples were kept at 4 °C until processing and analysed at the Parasitology Laboratory of the Department of Animal Pathology of the University of Las Palmas de Gran Canaria. *Eimeria* oocysts per gram of faeces (OPG) were quantified by a modified McMaster method (Bangoura and Daugschies 2007). In case of high OPGs, serial 1:10 dilutions were applied to facilitate counting.

For species differentiation and the estimation of the sporulation rate, the remaining faecal material was transferred to Petri dishes (Nunc) to allow for oocyst sporulation. Sporulation conditions and method for *Eimeria* species identification were as described above. This procedure was carried out for each of the faecal sampling days.

### Statistical analysis

Faecal oocyst counts per gram of stool (OPG) were log transformed into Log (OPG + 1) to obtain normal distributions (Kolmogorov-Smirnov normality test). For productive monitoring, apart from directly comparing body weights between groups at the different weeks of the experiment, the growth rates over time were estimated as follows: ln weight 2-ln 1) / *t* × 100, where *t* represents the number of days between sampling times 1 and 2. Comparison of OPG counts among the different groups was performed by one-way repeated-measures analysis of variance. For all pairwise multiple comparison procedures, the Holm-Sidak method was used, while the Krustal-Wallis test was employed for faecal score analysis. The average faecal consistency score per animal and the average of log (OPG+1) per animal were calculated and then compared among the four groups by one-way ANOVA with pairwise post hoc comparisons by the Tukey test. The software SigmaPlot 14.5 was employed for the analyses and differences were considered significant at a *P* <0.05 level.

## Results and discussion

The results of the present work show that immunization with attenuated oocysts from a mixture of *Eimeria* species could be an alternative for the control of caprine coccidiosis. Overall, the protective immune response mounted in the immunized goat kids led to a reduction of oocyst production by more than 80% (*P* < 0.05) during challenge infection. Additionally, a slight amelioration in clinical signs and a moderate increase in growth rates were detected in comparison to non-immunized challenge controls. The degree of protection achieved equals that reported in chickens being immunized against *Eimeria tenella* (Jenkins et al. 1991) or *Eimeria maxima* (Jenkins et al. 1997) when using ionizing radiation for oocyst attenuation.

**Fig. 1:**
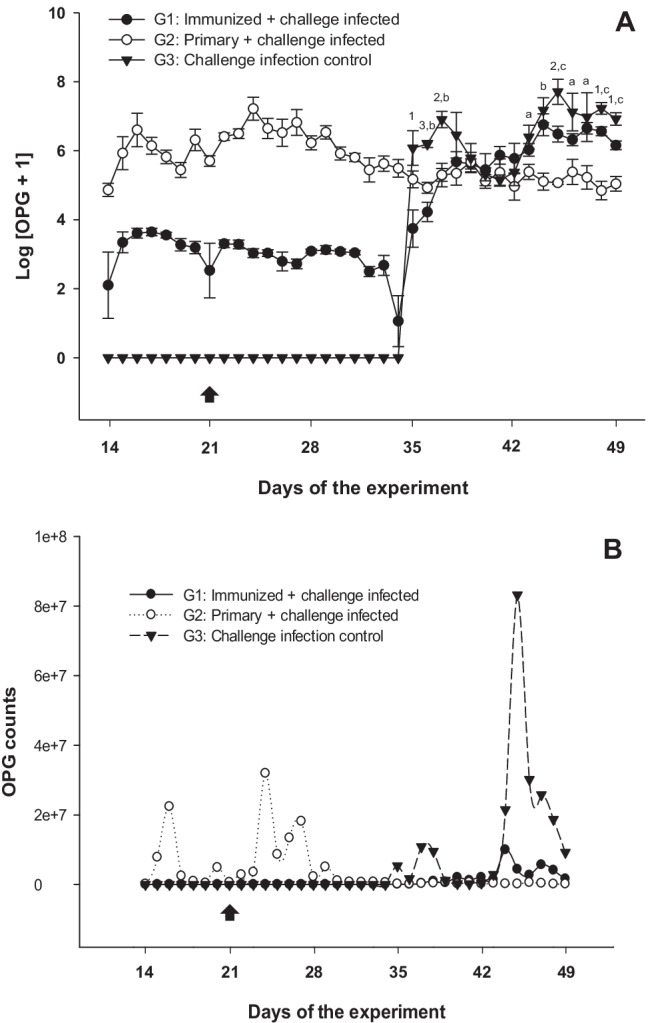
Log-transformed *Eimeria* oocyst counts per gram of faeces [Log (OPG+1)] (**A**) and raw OPG counts (**B**) in immunized goat kids and related control groups. The data are represented by mean ± SEM (**A**) or only mean values (**B**). For statistical differences: (1, 2, 3) *P* < 0.05 - *P* < 0.01 - *P* < 0.001 G1 vs G3; (a, b, c) *P* < 0.05 - *P* < 0.01 - *P* < 0.001 G2 vs G3. Day of challenge infection is indicated by an arrow

As expected, goats immunized with attenuated oocysts (G1) showed significantly lower OPGs during primary infection (days 14–35 of the experiment) than those infected with non-attenuated oocysts (G2) (*P* < 0.001) (Fig. 1A), thereby proving the effectiveness of the attenuation. Furthermore, the inoculum was suitable to induce acquired immunoprotective response and, consequently, to reduce oocyst shedding after challenge infection when comparing to challenge infection control (significant reactions on days 35, 36, 37, 45, 48 and 49 p. i., with *P* < 0.05 to *P* < 0.001). Non-log-transformed faecal counts showed huge individual variations within the same group, particularly around peak values, which hampered a reliable statistical analysis, but more clearly illustrated the magnitude of oocyst reduction in sensitized goat kids (G1 and G2) compared to challenge control G3 (Fig. 1B). Based on these data, oocyst reduction levels between 53.6% (44 d p. i.) and 99.2% (35 d p. i.) were achieved in G1 depending on the day of sampling. The effects on oocyst production were more evident when mean cumulative OPG counts were compared between groups, taking cumulative counts as the sum of individual OPG values at all sampling times. Thus, an overall reduction of oocyst shedding of 81.9% (*P* <0.05) was calculated in group G1 compared to challenge controls (G3) during the reinfection phase (days 35–49 of the experiment). In line with current findings, the level of immunoprotection achieved by administration of attenuated oocysts in avian coccidiosis was also strongly associated to a significant reduction in oocyst excretion (Crouch et al. 2003). The reduction in cumulative overall oocyst production was higher in primary and challenge infected animals with non-attenuated oocysts (G2: 97.5%, *P* <0.05), but when considering oocyst shedding during both primary and reinfection phases (days 14–49 of the experiment), G1 showed reduction of the overall cumulative oocysts release of 76.8% (*P* <0.05) when compared to G2. In general, the total amount of oocysts released to the environment is of high epidemiological value, as it constitutes the main source of infection in oral-faecally transmitted diseases, such as coccidiosis.

**Fig. 2:**
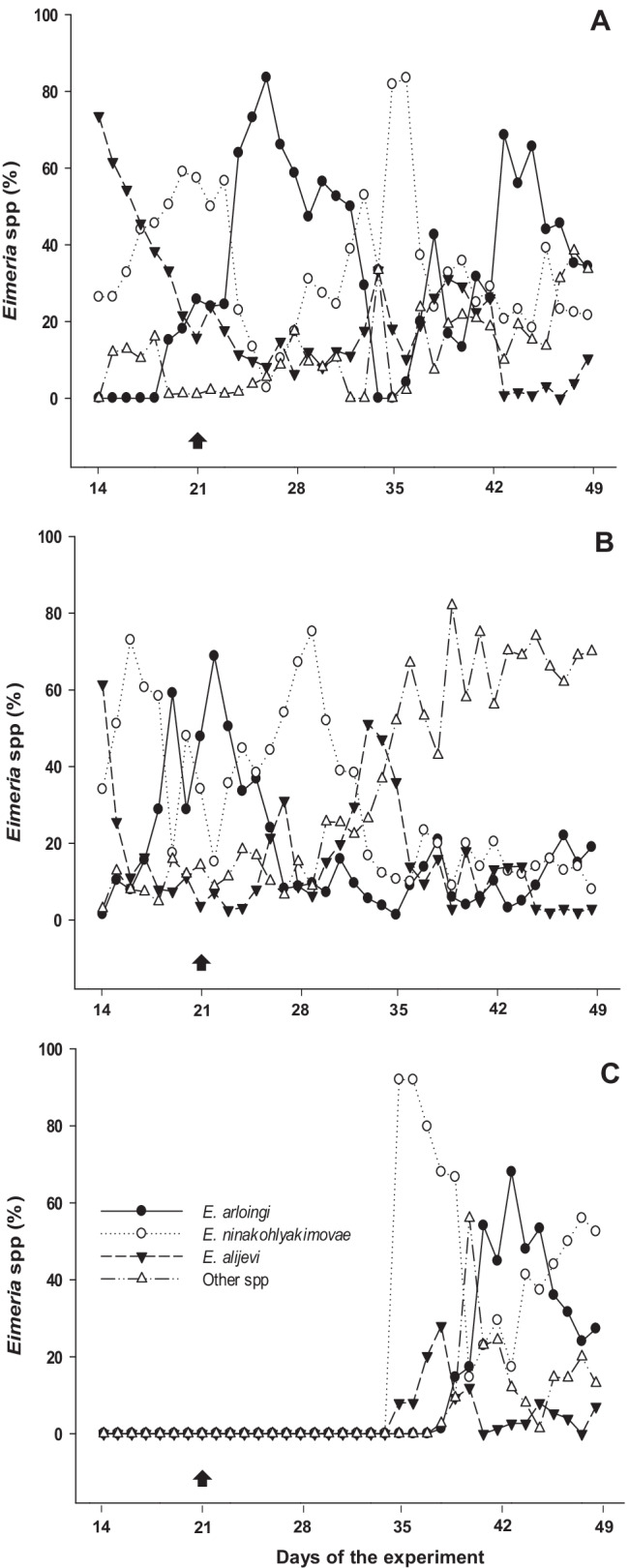
Percentage of the *Eimeria* species found in faeces of immunized goat kids and related control groups: **A** “G1: Immunized + challenge infected”; **B** “G2: Primary + challenge infected”; **C** “G3: Challenge infection control”. Day of challenge infection is indicated by an arrow

In total, 9 caprine *Eimeria* species were initially identified in immunization doses; the majority of the oocysts corresponded to *E. arloingi* (63.2%) followed by *E. ninakohlyakimovae* (19.7%), with minor species being *E. alijevi* (7.6%), *E. caprina* (5.5%), *E. christenseni* (2.2%), *E. hirci* (1.1%), *E. caprovina* (0.4%), *E. aspheronica* (0.2%) and *E. jolchijevi* (0.1%). The composition of *Eimeria* spp. oocysts shed by G1–G3 upon infection is presented in Fig. 2A–C. The analysis of the data confirmed that the major species were the same as in the initial inoculum, even though differences between groups occurred. Referring to kinetics of *Eimeria* species of animals immunized with attenuated oocysts (G1) (Fig. 2A), *E. alijevi* oocysts were the first to be shed (72% already in the first days of the experiment), followed by *E. ninakohlyakimovae* (up to 60% on day 20) and *E. arloingi* (more than 80% on day 26). The current kinetics agrees with known prepatency of these *Eimeria* species (Ruiz et al. 2013a; Silva et al. 2014b; Ruiz and Molina 2019). In the course of the challenge infection, approximately the same sequence was observed, but peak values were lower and shorter than during primary infection, indicating that immune response driven by attenuated oocysts was effective against the three more frequent *Eimeria* species (Fig. 2A). In the case of *Eimeria* species of poultry, it has been reported that pathogenic species are often bearing low immunogenic capacities (Jacobs et al. 2016); however, the current data would indicate that, in the caprine system, attenuated oocysts induce protection in both pathogenic and non-pathogenic *Eimeria* species. Primary infected goat kids with non-attenuated oocysts (G2) shared same profiles as group G1, with *E. alijevi*, *E. ninakohlyakimovae* and *E. arloingi* being the main species shed soon after infection (Fig. 2B). However, after challenge infection, some changes were observed compared to group G1 since, particularly at the end of the experiment, percentages of *E. ninakohlyakimovae* and *E. arloingi* were significantly low, indicating that a strong protective immune response against these two *Eimeria* species indeed was developed. Discrepancy with respect to what previously described for G1 could be explained by a higher exposure of G2 to full viable parasites during primary infection (Fig. 1) as described in poultry coccidiosis (Blake et al. 2005), where the immunity developed against *E. maxima* was strongly influenced by the immunizing dose size. Finally, the same pattern was confirmed for the challenge control group (G3), except that *E. ninakohlyakimovae* but not *E. alijevi* was the most predominant *Eimeria* species at the beginning of oocyst shedding (Fig. 2C). The last difference may be related to the age of the animals at the time of infection (which was 3 weeks later than in G1/G2), as it has been previously suggested from experimental *Eimeria* infections performed in goat kids of different age (Matos et al. 2018).

No differences were found when comparing the sporulation rate of oocysts coming from goat kids infected either with attenuated or not attenuated oocysts (mean values of about 95% in all groups). This would mean that attenuated oocysts able to complete their endogenous cycle may release viable oocysts. However, additional studies are needed to investigate whether these oocysts, in addition to sporulating, have the capacity to continue the endogenous cycle upon infection. If so, as suggested previously (Ruiz et al., 214), new released oocyts would ensure further boosting of the immunity within the herd.

**Fig. 3:**
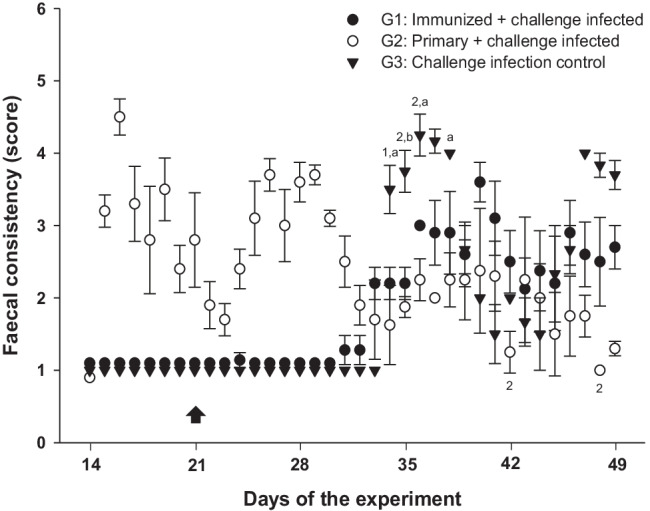
Faecal consistency in immunized goat kids and related control groups. The following score was used: (1) normal formed faeces; (2) unformed faeces of slightly reduced consistency; (3) faeces of moderately reduced consistency; (4) yellowish, greenish or brownish liquid faeces; (5) liquid faeces of reddish colour and/or presence of mucosa pieces. Data are expressed as means ± SEM. For significant differences: (1, 2, 3) *P* < 0.05 - *P* < 0.01 - *P* < 0.001 G1 vs G3; (a, b, c) *P* < 0.05 - *P* < 0.01 - *P* < 0.001 G2 vs G3. Day of challenge infection is indicated by an arrow

Clinical coccidiosis in goats usually coincides with destruction of the gut mucosa during gametogony, which approximately takes place 2 weeks after infection (Dai et al. 2006; Ruiz et al. 2013a). Correspondingly, primary infected animals with non-attenuated oocysts (G2) showed severe diarrhoea, with scores ranging between 3 and 4 within days 15–32 of the experiment; from this point onwards, the faecal consistency improved to normal within the last 2 weeks of the experiment (Fig. 3). Similar findings applied to primary infected challenge controls (G3, Fig. 3). In contrast, animals immunized with attenuated oocysts (G1) showed normal stool consistency during primary infection, therefore complying with one of the main requirements of a vaccine candidate, requesting that vaccination itself should not induce considerable disease. However, challenge infection had slight effects on G1-related faecal consistency (especially at 34–43 days, Fig. 3) indicating that immunization did not fully prevent clinical coccidiosis. This finding is consistent with the OPG counts and with the presence in this group of *E. ninakohlyakimovae* and *E. arloingi*, representing the two most pathogenic *Eimeria* species of goats (Ruiz et al. 2013a, b; Silva et al. 2014b). Nevertheless, faecal consistency was closer to normal on most days of analysis when compared to challenge controls (G3), i. e. at days 35–39 of the experiment (= 14–18 days p. i.) (*P* < 0.05 to *P* < 0.01). The same held true for goat kids primary and challenge infected with non-attenuated oocysts (G2) (Fig. 3).

**Fig. 4:**
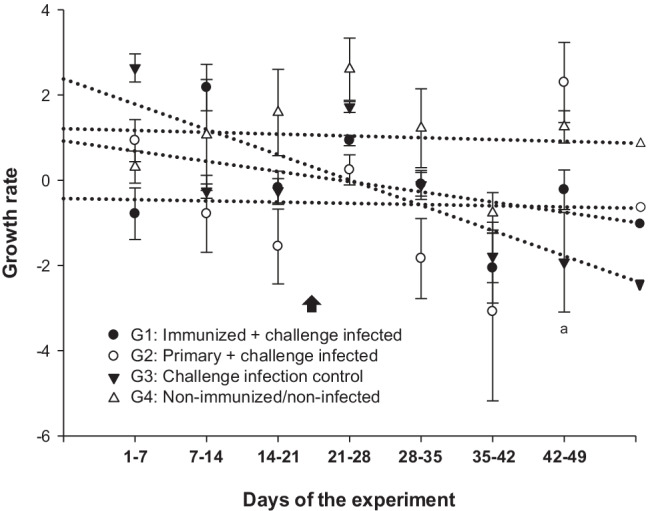
Growth rates in immunized goat kids and related control groups. The data are expressed as means ± SEM. For significant differences: (a) *P* < 0.05 G2 vs G3. Day of challenge infection is indicated by an arrow

Commonly, coccidiosis leads to considerable economic losses based on reduced productive parameters and animal losses. In line, challenge controls (G3) showed a decrease of growth rates when compared to non-infected animals (G4). Different studies linked immunoprotection against avian coccidiosis to improved productive parameters (Jenkins et al. 1991, 1997; Crouch et al. 2003). For the caprine system, a comparable effect was not confirmed in recent studies (Ruiz et al. 2014). Likewise, we failed to demonstrate a significant effect of immunization on growth rates in the present study even though temporary improved growth rates were detected within a short time frame of the experiment (42–49 days; G2 vs G3: *P* < 0.05) (Fig. 4). Thus, as for group G3, animals immunized with attenuated oocysts and challenged (G1) showed a negative growth-related slope during the last 2 weeks of the experiment, which coincide with the presence of clinical coccidiosis during the reinfection phase. Body weight impairment is also a common finding in ruminant coccidiosis (Alyousif et al. 1992; Daugschies et al. 2007; Ruiz et al. 2013a).

These results presented here are partially in line with those reported by Ruiz et al. (2014), where a similar immunization protocol was used but investigating monospecific immunization against *E. ninakohlyakimovae*. In the latter study, immunization with attenuated oocysts prevented immunized animals from severe clinical coccidiosis during both primary and challenge infections and the level of immunoprotection achieved in terms of reduced oocyst production was higher than the one found here. These differences could be related to the complexity of the interactions established between the different species of *Eimeria* included in the inoculum, which could have resulted in (1) higher oocyst counts, (2) more severe clinical signs of coccidiosis, and (3) more prolonged patency; despite the fact that the infecting dose of oocysts was exactly half of that used by Ruiz et al. (2014) (1 × 10^5^ vs 2 × 10^5^ sporulated oocysts). On the other hand, the use of a lower dose of immunogen could also be related to the lower degree of immunoprotection found in the present study, probably insufficient to trigger a solid effective immune response in the case of pathogenic species *E. ninakohlyakimovae* and *E. arloingi*; indeed, as described previously for poultry (Blake et al. 2005), the elicited immunity is strongly influenced by the immunizing size. Other factors, such as the age of the animals at the time of the immunization or the dose of irradiation used to attenuate the oocysts, could be standardized in order to implement the efficacy of the vaccine. Regarding the latter, an interesting study has been recently published showing that *Eimeria tenella* oocysts attenuated by low-energy electron irradiation (LEEI) induce protection against challenge infection in chicken (Thabet et al. 2019). As a whole, the preliminary immunization proposal outlined in the present study would require additional optimization approaches that improve immunoprotection and the corresponding impact on productive parameters. To this purpose, we are currently designing on-farm field studies for the evaluation of a greater number of variables in relatively large group sizes. As mentioned above, these parameters will include the age of the animals at the time of immunization, the infecting dose, the degree of attenuation of the oocysts, among other factors.

So far, the immunological mechanisms mediating immunoprotection against caprine coccidiosis by the current immunization protocol remain unclear. Most likely, attenuated oocysts would be able to excyst in vivo and resulting sporozoites could still invade epithelial/endothelial cells. However, some of these sporozoites may lack further or complete development, finally resulting in less or smaller meronts which still are able to stimulate immune responses. This hypothesis would explain reduced OPG counts and lower intensities of clinical signs observed in the current study. The hypothesis that acquired immune responses can already be triggered during the first merogony is consistent with observations made by our group showing that the number of immature schizonts found in the pre-patent period (7 days p. i, merogony I) was significantly lower in primary and subsequently challenged *E. ninakohlyakimovae* infected animals compared to challenge controls (Matos et al. 2017b). This assumption is in line with reports showing that immunization of chicken with irradiated *E. tenella* oocysts confers protection that does not require full development of first-generation meronts during primary infection (Jenkins et al. 1991).

## Conclusions

The present work constitutes the first evidence of immunoprotection induced in ruminants against coccidiosis by using oocysts from a mixture of *Eimeria* species being attenuated by ionizing X irraditation. The degree of immunoprotection in terms of reduced faecal oocyst shedding and improvement of clinical disease suggests this type of immunization for future caprine/ruminant coccidiosis control. As a prerequisite, several factors should be further investigated, including (i) the age of the animals at the moment of immunization; (ii) the immunizing dose size; (iii) the degree and technique of oocyst attenuation; and (iv) a detailed study of interactions between different *Eimeria* spp.

## Data Availability

All data presented here are available upon request.
